# High-Resolution Separation of Nanoparticles Using a Negative Magnetophoretic Microfluidic System

**DOI:** 10.3390/mi13030377

**Published:** 2022-02-26

**Authors:** Lin Zeng, Xi Chen, Rongrong Zhang, Shi Hu, Hongpeng Zhang, Yi Zhang, Hui Yang

**Affiliations:** 1 Laboratory of Biomedical Microsystems and Nano Devices, Bionic Sensing and Intelligence Center, Institute of Biomedical and Health Engineering, Shenzhen Institute of Advanced Technology, Chinese Academy of Sciences, Shenzhen 518055, China; lin.zeng@siat.ac.cn (L.Z.); xi.chen@siat.ac.cn (X.C.); zhangrr@siat.ac.cn (R.Z.); shi.hu@siat.ac.cn (S.H.); 2 Marine Engineering College, Dalian Maritime University, Dalian 116026, China; zhppeter@dlmu.edu.cn; 3 Center for Medical AI, Institute of Biomedical and Health Engineering, Shenzhen Institute of Advanced Technology, Chinese Academy of Sciences, Shenzhen 518055, China; yi.zhang3@siat.ac.cn

**Keywords:** negative magnetophoresis, nanoparticles, separation, microfluidic chip

## Abstract

The separation and purification of a sample of interest is essential for subsequent detection and analysis procedures, but there is a lack of effective separation methods with which to purify nano-sized particles from the sample media. In this paper, a microfluidic system based on negative magnetophoresis is presented for the high-resolution separation of nanoparticles. The system includes on-chip magnetic pole arrays and permalloys that symmetrically distribute on both sides of the separation channel and four permanent magnets that provide strong magnetic fields. The microfluidic system can separate 200 nm particles with a high purity from the mixture (1000 nm and 200 nm particles) due to a magnetic field gradient as high as 10,000 T/m being generated inside the separation channel, which can provide a negative magnetophoretic force of up to 10 pN to the 1000 nm particle. The overall recovery rate of the particles reaches 99%, the recovery rate of 200 nm particles is 84.2%, and the purity reaches 98.2%. Compared with the existing negative magnetophoretic separation methods, our system not only exhibits high resolution on particle sizes (800 nm), but also improves the sample processing throughput, which reaches 2.5 μL/min. The microfluidic system is expected to provide a new solution for the high-purity separation of nanoparticles, as well as nanobiological samples.

## 1. Introduction

Nanobioparticles such as viruses, subcellular organelles, extracellular vesicles, etc., have shown their important roles in disease diagnosis and treatment in recent years, but there is a lack of efficient methods with which to separate and extract nanobioparticles with high purity [1]. Microfluidic devices have become a popular tool for biological sample processing and analysis due to their small size, fast response, low cost, and feasibility to develop into highly integrated portable devices [2]. Since the critical dimension of a microfluidic channel is close to that of micrometer-sized particles, such as cells, a microfluidic device therefore shows advantages that are usually difficult to achieve through traditional technology in the separation and detection of microparticles [3,4]. To date, microfluidic-based separation techniques using magnetic field [5,6], surface acoustic wave [7], dielectrophoresis [8], optics [9], inertia effect [10], and deterministic lateral displacement [11], etc., have become technically mature in cell separation. However, when these techniques are applied to separate nanoparticles, good results can hardly be achieved [12]. Various methods have been proposed to separate and purify nanoparticles [13,14], but some of them would cause damage to biological samples, for example, due to the Joule heat generated by the optical field [15] and the surface potential generated by dielectrophoresis [16].

Among various techniques, magnetic separation provides a relatively more straightforward solution that would not bring damage to biological samples, and the magnetic field is flexible and controllable [17,18]. Magnetic separation can be categorized into labeled and label-free methods based on the different methods of processing biological samples, corresponding to positive magnetophoresis and negative magnetophoresis, respectively. Positive magnetophoresis requires magnetic beads to label the target biosamples, and therefore the biosamples can be precisely controlled by an external magnetic field, however, in this case, requiring magnetic beads as the solid substrate to capture the biological samples and additional elution protocols [19,20]. Negative magnetophoresis has been recently proposed for cell separation and has drawn much attention due to its label-free nature. By using paramagnetic salt solution or ferrofluid as the sample medium, negative magnetophoresis can separate the non-magnetic biosamples based on their size difference [21,22]. However, the size resolution, i.e., the minimum difference of the sizes of the biosamples that can be separated from a mixture state often remains at the micrometer-scale, since permeability of the magnetic solution and magnetic field gradients are usually very low, therefore the magnetic separation force is also small [23,24].

The development of ferrofluid-based negative magnetophoresis started from the use of external permanent magnets to separate microparticles and cells [25,26]. In order to improve the size resolution, other forces such as viscoelasticity [27], inertial force [28], and centrifugal force [29] are combined with the negative magnetophoretic force in a single chip. Recently, new magnetic structures have been used to improve the magnetic field gradient, for example, utilizing four permanent magnets presented by Mao et al. [30], as well as generating an on-chip magnetic pole array in our previously published work [31], both of which greatly improve the magnetic field gradient and successfully achieve a separation of 200 nm and 1000 nm particles, however the sample throughput in these two works is not high enough.

Here, we present a new microfluidic system for the separation of nanoparticles based on negative magnetophoresis. With the unique design of the microfluidic and magnetic structures in the system, nanometer-sized particles can be efficiently separated with high purity and high sample throughput.

## 2. Materials and Methods

### 2.1. Chip Design

**Figure 1:**
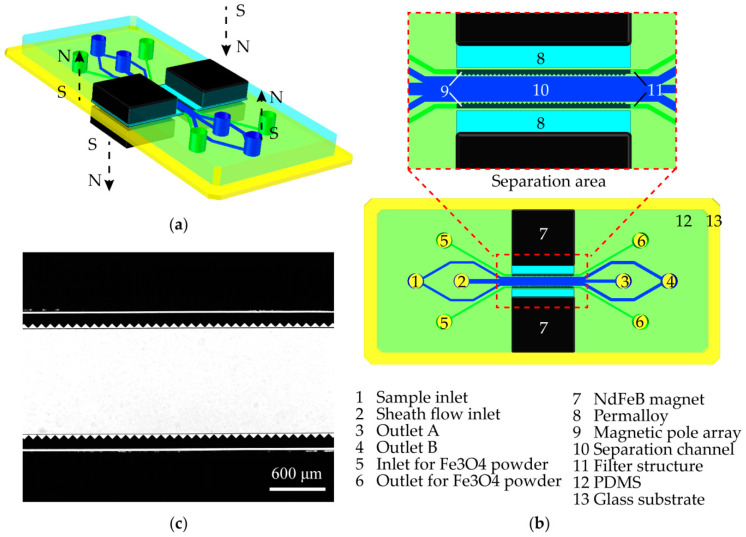
Design and image of the microfluidic system: (**a**) Overall structure of the system; (**b**) Schematic illustration on the system design; (**c**) Microscopic image on the separation area.

Details on the chip design are shown in Figure 1. The system consisted of four external permanent magnets, two high-permeability alloys (permalloy, 1J85), two on-chip magnetic pole arrays, and a separation channel. The external permanent magnets provide a strong magnetic field, which can be conducted to the on-chip magnetic pole arrays through the high-permeability alloys, then the magnetic pole arrays arranged on both sides of the separation channel can generate a high-gradient and high-intensity magnetic field in the separation channel. The ferrofluid, which is made up of Fe_3_O_4_ nanoparticles (10 nm) suspended in water, generated a concentration difference in the separation channel; the higher the magnetic field gradient is, the higher the concentration of the Fe_3_O_4_ nanoparticles, therefore the non-magnetic particles suspended in the ferrofluid are subjected to a pressure that is caused by the concentration difference of the Fe_3_O_4_ nanoparticles, which is referred to as the “negative magnetophoretic force”, and the direction towards to the low magnetic field gradient area. 

In this chip, the sheath fluid can focus the sample particles on both sides of the separation channel (that is, the area close to the magnetic pole arrays). The 200 nm particles can flow into the outlet channels on both sides and finally flow into outlet B. The magnetic field gradient and magnetic field strength in the side area are relatively high, which can make the 1000 nm particles deflect toward the center of the separation channel by a strong negative magnetophoretic force, and the 1000 nm particles finally flow into outlet A. Thereby, particles with different sizes can be separated and collected from the different outlets of the microfluidic system.

### 2.2. Fabrication of the Microfluidic System

The fabrication of the system was divided into two steps. The first step was to fabricate a conventional microfluidic chip using soft lithography. The height of all microchannels was 50 μm, the width of the two sample inlet channels was 100 μm, the width of the sheath flow inlet channel was 1000 μm, the width of the separation channel was 1200 μm (as shown in Figure 1b), and the width of three outlet channels at the end was 400 μm. The second step was to build a high-gradient magnetic field structure on the chip. The Fe_3_O_4_ powders (5–10 μm in diameter) mixed with ethanol were first injected into the magnetic pole channels to form the magnetic pole arrays. The width of the magnetic pole channels on both sides was 100 μm, and the distance from the tip of the magnetic pole to the separation channel was 10 μm. Then, the permalloy (1J85, 30 μm in thickness, Dongguan Saijing Special Alloy Co., Ltd., Dongguan, China) was embedded in both sides of the magnetic pole arrays; here, we reserved a permalloy embedded channel (26 mm in width and 50 μm in height) on the sides of the magnetic pole arrays, so that the rectangular permalloy could be precisely embedded in both sides of the magnetic pole arrays under the microscope. Finally, the magnets (N52, 25.4 mm × 12.7 mm × 3.175 mm) were added to the chip through a fixed mold. The distance from the permalloy to the magnetic pole channel was 30 μm, and the length of the magnetic pole array was 26 mm.

### 2.3. Experimental Setup

In order to verify the effect of the separation system, fluorescent polystyrene particles (non-magnetic) with sizes of 200 nm (yellow-green, Thermo Fisher Scientific, ~35,000 particles/μL) and 1000 nm (glacial blue, Bangs Laboratories, Inc, Fishers, IN, USA., ~3500 particles/μL) were mixed in the diluted ferrofluid (EMG 805, 3.6%). First, the ratio setting was chosen to mimic the concentration ratio of micro-extracellular vesicles (microEVs, >800 nm) and small-extracellular vesicles (sEVs, <200 nm) in blood plasma [13], since the sEVs are the typical nanobiological samples which may be separated using our system in the future. When the concentration of the 1000 nm particle increases, the interaction between the two kinds of the particles increases as well. On one hand, it may slightly affect the separation performance; on the other hand, the impact is extremely limited based on the previous reference [24]. Then, the mixture sample and the sheath fluid (diluted ferrofluid without fluorescent particles) were injected into the chip. In the experiments, the sample concentration and flow rate were optimized first. Fluorescence trajectories of the particles were recorded using an inverted microscope (Zeiss Axio Observer 7, Carl Zeiss Microscopy GmbH, Jena, Germany) equipped with appropriate fluorescent filter sets and an sCMOS camera (ORCA-Flash4.0-V3, Hamamatsu, Japan). Then, we used the optimal parameters to separate the fluorescent particles. The separation efficiency and the recovery rate of 200 nm particles were then analyzed using a flow cytometer (FCM, CytoFLEX S, Beckman Coulter, Atlanta, GA, USA).

## 3. Results

We first analyzed the force acting on the particles in the microfluidic channel, and the magnetic field of the chip and the particle trajectories were simulated using the finite element method in Comsol Multiphysics, and finally the separation experiments were carried out to verify the performance of the system.

### 3.1. Theory and Mechanism

**Figure 2:**
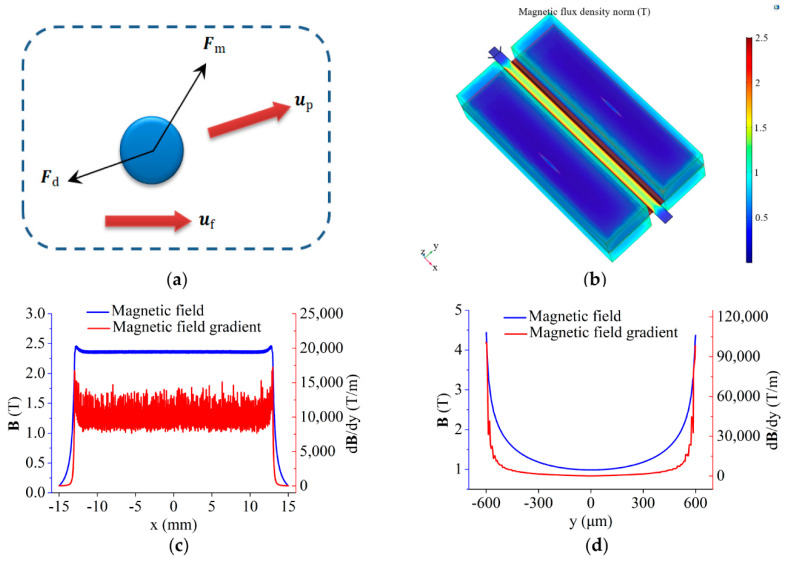
Simulation results of the magnetic field and particle trajectories: (**a**) The force analysis of the particle in separation channel; (**b**) The magnetic field distribution of separation area; (**c**) The magnetic field and its gradient in separation area along *x*-axis; (**d**) The magnetic field and its gradient in separation area along *y*-axis.

When particles are transported through the microchannel, they flow forward under the hydrodynamic drag force $F_{d}$, and the negative magnetophoretic force $F_{m}$ acts on the particles when they enter the separation area, as shown in Figure 2a. The hydrodynamic drag force $F_{d}$ is described by the following equation [32]:

(1) $$F_{d}=3\pi \eta_{f}D_{p}f_{D}\left(u_{f}-u_{p}\right)$$

where $\eta_{f}$ is the dynamic viscosity of the ferrofluid, $D_{p}$ is the diameter of the particle, $u_{f}$ and $u_{p}$ are velocity vectors of ferrofluid and particle, respectively. $f_{D}$ is the hydrodynamic drag force coefficient that is determined by the structure of microchannel and the particle position:

(2) $$f_{D}={\left[1-\frac{9}{16}\left(\frac{D_{p}}{D_{p}+2L}\right)+\frac{1}{8}{\left(\frac{D_{p}}{D_{p}+2L}\right)}^{3}-\frac{45}{256}{\left(\frac{D_{p}}{D_{p}+2L}\right)}^{4}\;-\frac{1}{16}{\left(\frac{D_{p}}{D_{p}+2L}\right)}^{5}\right]}^{-1}$$

where L is shortest distance between the surface of the particle and the channel wall.

The negative magnetophoretic force $F_{m}$ is described by the following equation [33]:

(3) $$F_{m}=\frac{V_{p}\left(\chi_{p}-\chi_{f}\right)}{\mu_{0}}\left(B\cdot \nabla \right)B$$

where $V_{p}$ is the volume of the particle, $\mu_{0}$ is the permeability of free space, B is the magnetic induction, $\chi_{p}$ and $\chi_{f}$ are the magnetic susceptibilities of particle and ferrofluid, respectively. The negative magnetophoretic force $F_{m}$ is proportional to the particle volume.

The particles of different sizes eventually enter different outlets following different trajectories that are determined by these two forces. The motion control equation is:

(4) $$m_{p}\frac{du_{p}}{dt}=F_{m}+F_{d}$$

Based on the Equations (1)–(4), the particle trajectories can be calculated by the simulation software. The initial velocity $u_{f}$ is set according to the experimental setup.

### 3.2. Simulation

Based on the chip design, a model of the separation area is established in Comsol Multiphysics. In simulation, the “Magnetic fields, No Current” module was used to solve the magnetic field and was applied to all domains; the “Laminar Flow” module was used to solve the flow field and was applied to the separation channel; and the “Particle Tracing for Fluid Flow” module was used to solve the particle-moving trajectories in ferrofluid and was also applied to the separation channel. The parameters used in the simulation are shown in Table 1 [34]. Here, the wall of the separation channel was set to no slip. The free tetrahedral mesh of all domains was predefined as finer. While in the separation channel domain, the mesh is refined to extremely fine, the maximum element size is set to 35 μm, and the minimum element size is set to 3 μm. The simulation result of the magnetic field is shown in Figure 2b. In the separation channel, we analyzed the magnetic field and its gradient along *x*-axis (y = 50 μm and z = 0 μm) and y-axis (y = 50 μm and z = 0 μm); as shown in Figure 2c,d, the origin of the coordinates is at the center of the separation channel. The simulation results show that the magnetic pole arrays in both sides can generate a magnetic field exceeding 2 Tesla (T) in the separation channel, and the highest magnetic field gradient reaches 10,000 T/m.

**Figure 3:**
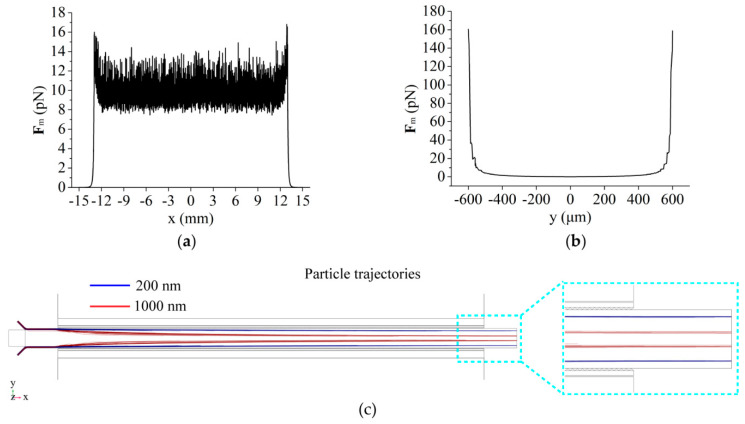
Calculated negative magnetophoretic force and particle trajectories based on the simulation results: (**a**) Negative magnetophoretic force acting on 1000 nm particles in the separation area along *x*-axis and (**b**) along *y*-axis; (**c**) The trajectories of 200 nm and 1000 nm particles in the simulation.

We calculated the negative magnetophoretic force on 1000 nm particles in the separation area based on the magnetic field simulation results and Equation (1), as shown in Figure 3a,b. When they flow through the separation channel along the channel side wall, the force of 1000 nm particles can reach 10 pN, which is strong enough to separate them from the mixture state.

Based on the magnetic field simulation and negative magnetophoretic force calculation, we further simulated the trajectories of 200 nm and 1000 nm particles (10 particles/s for each size) in the separation area, as shown in Figure 3c. The negative magnetophoretic force is used to drive the 1000 nm particles, pushing them further away from both sides of the separation channel and eventually moving into outlet A. The results show that the negative magnetophoresis plays a key role in separating the 1000 nm particles. Since the negative magnetophoretic force is proportional to the particle volume, the deflection distance of the 200 nm particles is therefore very small, and most of the 200 nm particles can be collected from outlet B. In order to achieve a high recovery rate of 200 nm particles, the dividing line of the two kinds of particles should be set close to the trajectories of 1000 nm particles (here, the width of three outlet channels at the end is 400 μm).

### 3.3. Separation Experiments

**Figure 4:**
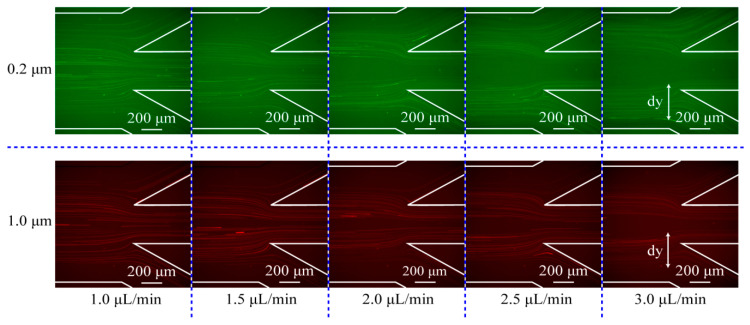
The trajectories of 200 nm and 1000 nm particles at different flow rates in the experiments.

In the experiments, the optimal concentration of the ferrofluid was chosen as 0.003× (0.01%), based on our precious study [31]. Here, we first optimized the flow rate of the sample, the results of which are shown in Figure 4. Compared with the simulation results, the trajectories in the experiments are more divergent. For both the 200 nm and 1000 nm particles, the trajectories are moving to the side channel as the flow rate increases. In order to optimize the flow rates, we calculated the position distribution of particle trajectories along the *y*-axis direction, which is defined here as dy.

**Figure 5:**
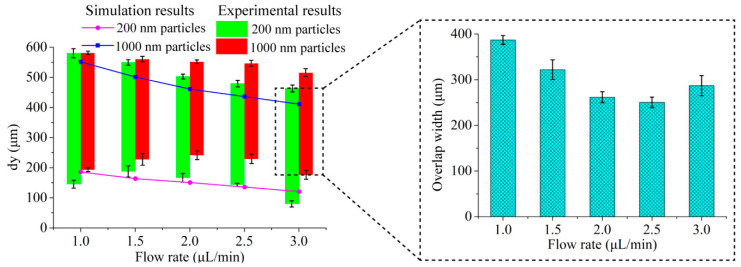
Comparison between simulation results and experimental results on particle distribution along *y*-axis at the outlet area under different flow rates of the sample flow.

The comparison between simulation results and experimental results on the position distribution of particle trajectories at the outlet area under different flow rates of the sample flow are shown in Figure 5. Based on the position distribution, we calculated the widths of the overlap area of the two kinds of particles at different flow rates, and the overlap area demonstrates that the two kinds of particles are not separated effectively. The results show that the overlap width is the shortest at a flow rate of 2.5 μL/min, therefore we can conclude that the optimal sample flow rate is 2.5 μL/min (the ratio of the flow rates of the sample flow and the sheath flow is kept as 1:5, the ratio set can ensure that the particles are focused close to the channel wall without dispersing to outlet A when there are no magnets). Under the optimal conditions, most of the 200 nm particles flow into side outlets and finally converge on outlet B. On the contrary, most of the 1000 nm particles flow into outlet A through the center channel (Figure 4).

It is noted that the simulation is performed in an ideal condition (the interactions between particles are ignored) and that the trajectories in the simulation are more concentrated than those in the experiments. 

**Figure 6:**
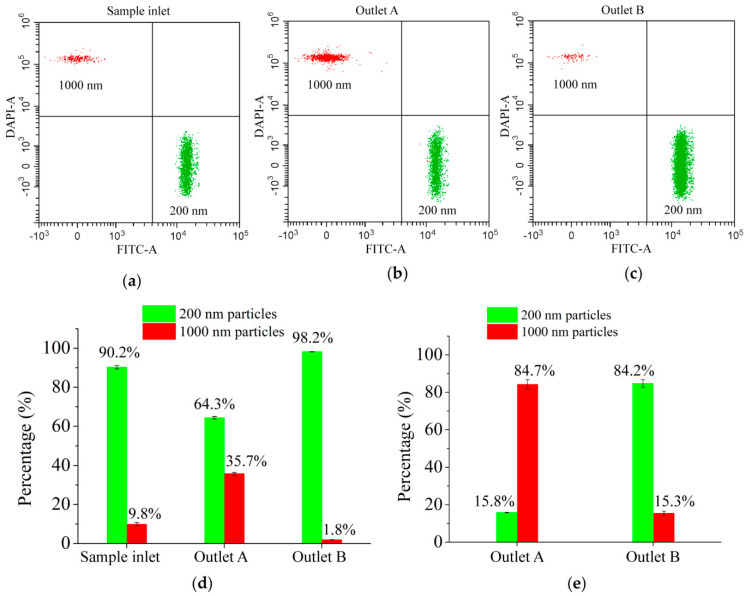
Flow cytometry test results obtained from: (**a**) Sample inlet; (**b**) Outlet A; (**c**) Outlet B; (**d**) The proportion of the two kinds of particles at inlet and outlets; (**e**) The number distribution of the two kinds of particles at both outlets.

In order to quantitatively analyze the particles collected from the two outlets, we used a flow cytometer to count the particles, the results of which are shown in Figure 6. Here, we used FITC and DAPI as the fluorescent detection channels for the 200 nm and 1000 nm particles, respectively, and the standard particles of both sizes were gathered in a cluster. The 1000 nm particles were detected in the upper left corner (red cluster), while the 200 nm particles were gathered in the lower right corner (green cluster). The overall recovery rate of all particles is 99.0% (the ratio of the number of particles from outlets to that from the inlet). The purity of 200 nm particles reached 98.2% (the ratio of the number of 200 nm particles to all the particles from outlet B) and recovery rate was 84.2% (the ratio of the number of 200 nm particles from outlet B to that from all outlets), and the resolution reached 800 nm. Using the optimal parameters, the chip can effectively separate 200 nm particles from the mixed particles of two sizes. Both the theoretical and experimental results prove the high performance of the proposed system.

## 4. Conclusions

In this paper, a microfluidic system for the high-resolution nanoparticle separation based on negative magnetophoresis is presented. By combining external magnets, permalloys, and on-chip magnetic pole arrays, the system can generate a magnetic field gradient as high as 10,000 T/m inside the separation microchannel, providing a negative magnetophoretic force (10 pN) that is large enough to separate 200 nm from the mixture samples (1000 nm and 200 nm). The recovery rate of 200 nm particles is 84.2% and the purity reaches 98.2%. The size resolution of our system reaches 800 nm and the throughput is improved to 2.5 μL/min. We believe that, in future, it may develop into a versatile tool to separate nanometric objects of environmental or biological importance, such as nanoparticles, viruses, or other biological agents.

## Figures and Tables

**Table 1:** The parameters used in the simulations.

Parameters	Value
Remanent flus density of the magnets	1.48 T
Relative permeability of ferrofluid after dilution	1.00069
Relative permeability of Fe_3_O_4_ powder	4
Relative permeability of permalloy	80,000

## Data Availability

The data that support the findings of this study are available from the corresponding author upon reasonable request.
